# Application of natural and modified additives in yogurt formulation: types, production, and rheological and nutraceutical benefits

**DOI:** 10.3389/fnut.2023.1257439

**Published:** 2023-10-30

**Authors:** Abigael Odunayo Bankole, Emmanuel Anyachukwu Irondi, Wasiu Awoyale, Emmanuel Oladipo Ajani

**Affiliations:** ^1^Department of Biochemistry, Kwara State University, Ilorin, Nigeria; ^2^Department of Food Science and Technology, Kwara State University, Ilorin, Nigeria

**Keywords:** yoghurt additives, yogurt types, yogurt processing, nutritional benefits, nutraceutical benefits

## Abstract

Yogurt, a popular fermented dairy product, is of different types and known for its nutritional and nutraceutical benefits. However, incorporating additives into yogurt has been adopted to improve its functionality and nutraceutical properties. Additives incorporated in yogurt may be natural or modified. The incorporation of diverse natural additives in yogurt formulation, such as moringa, date palm, grape seeds and argel leaf extracts, cornelian cherry paste, mulberry fruit and leaf powder, lentil flour, different types of fibers, lemongrass and spearmint essential oils, and honey, has been reported. Similarly, modified additives, such as β-glucan, pectin, inulin, sodium alginate, and gelatin, are also added to enhance the physicochemical, textural, sensory, and rheological properties of yogurt. Although additives are traditionally added for their technological impact on the yogurt, studies have shown that they influence the nutritional and nutraceutical properties of yogurt, when added. Hence, yogurts enriched with functional additives, especially natural additives, have been reported to possess an improved nutritional quality and impart several health benefits to consumers. These benefits include reducing the risk of cardiovascular disease, cancer, osteoporosis, oxidative stress, and hyperglycemia. This current review highlights the common types of yogurt, the production process, and the rheological and nutraceutical benefits of incorporating natural and modified additives into yogurt.

## 1. Introduction

Yogurt, a milk product and a prominent fermented dairy product, has become a popular and nutritious food option in the modern-day diet. It is more nutritious when compared to milk from which it is made (1). Similarly, its consumption has increased over time, due to its proven health and nutritional benefits (2). Yogurt is made by fermenting milk with bacteria, such as *Streptococcus thermophilus* and *Lactobacillus delbrueckii* subsp. *bulgaricus*, to form lactic acid (3). The lactic acid formed reduces the pH of milk, giving it a characteristic tangy taste and thick texture (4, 5).

The consumption of yogurt can be traced back to the Middle East herders, who carried milk in intestinal bags. The nomads soon discovered that the stored milk began to sour and cuddle, giving rise to the yogurt people enjoy today (6). In no time, yogurt gained ground throughout the Middle East and Europe. In the 16th century, the first yogurt factory was established in Barcelona, Spain. Currently, yogurt has been upgraded with different additives to enhance its sensory properties and influence its nutritional and nutraceutical properties (7). The nutritional composition of yogurt has made it a widely accepted dairy food product. It contains carbohydrates, proteins, minerals, and essential vitamins (8). Yogurt consumption has also been linked to several health benefits, including hastening digestion, which is attributed to its probiotic constituents, enhancing the gut’s microflora (9), and preventing the symptoms of constipation, bloating, and diarrhea (9).

Nutritionally, protein is one of the important nutrients in yogurt (10). The protein present in yogurt can be easily digested. Hence, it is a perfect diary option for individuals with impaired protein digestion (10). In addition, the consumption of yogurt has been reported to improve heart health (8, 9). This is because yogurt has proven to reduce cholesterol levels, thereby decreasing the danger of heart disease (9). Similarly, the presence of minerals in yogurt, calcium precisely, has proven to improve the cardiovascular health of its consumers. Studies have shown that the blood pressure of hypertensive patients, who consumed yogurt decreased significantly (11). In addition, yogurt was found to lessen the risk of type 2 diabetes by improving insulin sensitivity and glucose tolerance (11). Yogurt has also been linked with improved bone density, especially in women in their post-menopausal stage, thereby decreasing the risk of osteoporosis, due to its high mineral content (12).

Different additives, including natural and modified additives, are added to yogurt to enhance its physicochemical, textural, sensorial, and rheological properties. They are added to yogurt for a variety of technological purposes, including as stabilizers, gelling agents, flavorants, preservatives, thickeners, and colorants. For example, gelatin addition is considered good due to its gelling characteristics, traceable to its reversible coil-to-helix transition when cooling (13). Hence, during cold storage of yogurts, formation of gelatin triple helices at junction points enhances gelation and reduces whey loss through syneresis (14, 15). However, aside from their technological functions, studies have shown that they also influence the nutritional and nutraceutical properties of yogurt, when added. Diverse natural additives, such as moringa seed (16), date palm spikelets (17), extracts of grape seeds (18) and argel leaf (19), cornelian cherry paste (20), mulberry fruit and leaf powder (21), lentil flour (22), different types of fibers (23), lemongrass and spearmint essential oils (24), and honey (25), incorporated in yogurt formulation have been reported. Similarly, studies have shown that modified additives, such as β-glucan (26), low-methoxyl pectin (27), inulin, and agave fructans (28) are added in yogurt.

The objective of this review is to highlight common yogurt types, the production process of yogurt, and the rheological and nutraceutical benefits of incorporating natural and modified additives into yogurt.

### 1.1. Types of yogurt

**Figure 1:**
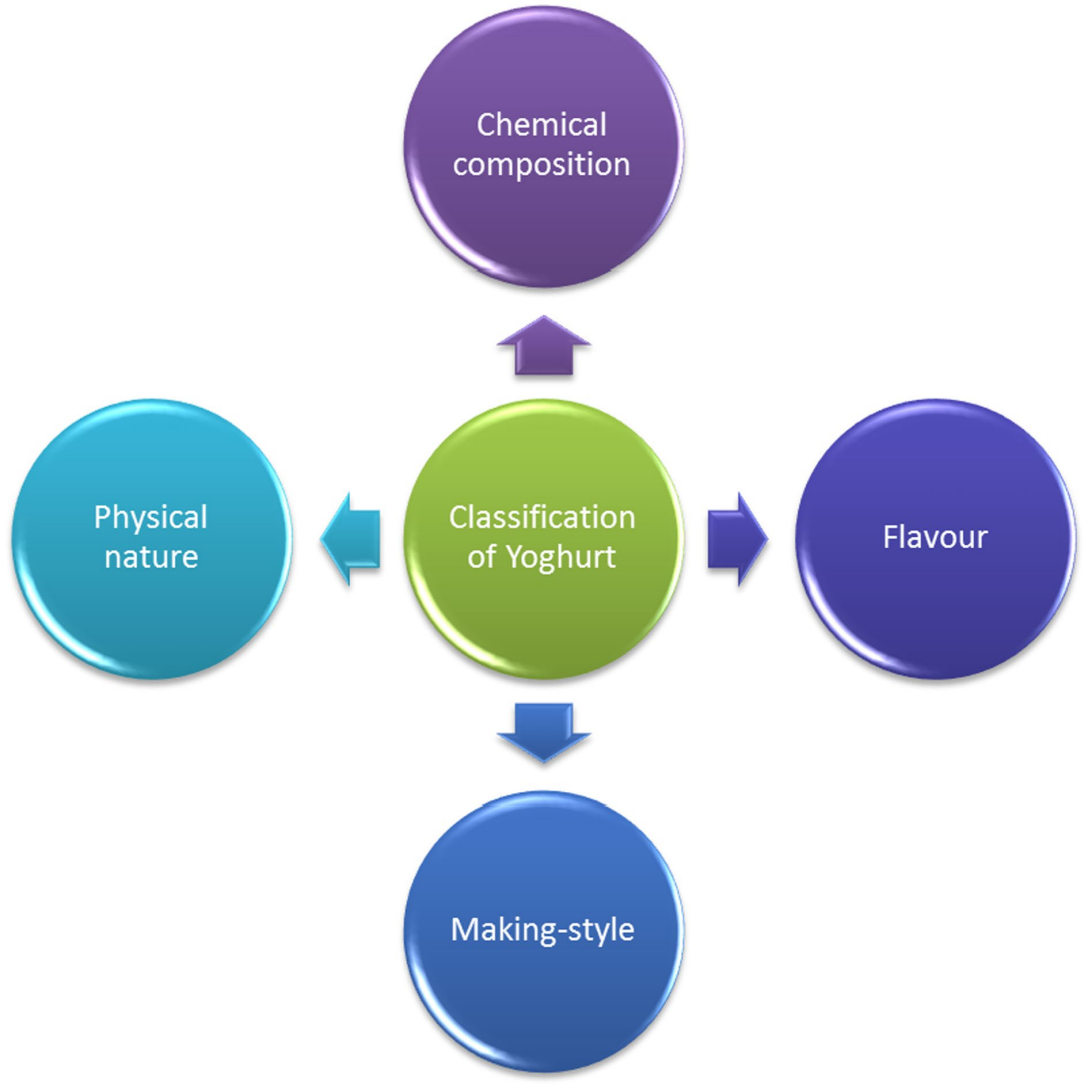
Classification of yogurt.

Yogurts are available in the market in a variety of textures, flavors, and forms that suit a wide array of meals and occasions. Several names are used to classify yogurt styles, some of which include live yogurt, traditional yogurt, stirred yogurt, set yogurt, yogurt drink, frozen yogurt, and Greek yogurt (29–31). Korkmaz et al. (32) claimed that the classification of yogurt is influenced by its preparation method, texture, pasting properties, and rheology (32). The parameters considered in classifying yogurt include its physical nature, chemical composition, making style/incubation process, and added flavors (Figure 1).

#### 1.1.1. Classification based on the physical nature

Yogurt can be fluid-like, semi-solid, or solid in nature. The set-type yogurt has a jelly-like solid texture and is often incubated and cooled in the final packaging stage (33). Yogurt that has a fluid nature is referred to as drinking yogurt. It goes through homogenization to reduce the particle size to ensure the stabilization of the protein suspension and hydrocolloid distribution (8). On the other hand, yogurt, in its semi-solid state, is referred to as stirred yogurt. It is often made by incubating the yogurt in a container, followed by pulverization by stirring, cooling, and packaging (34).

#### 1.1.2. Classification based on the chemical state/composition

Yogurt can be grouped into three major variations based on its fat content. They include regular yogurt, non-fat yogurt, and low-fat yogurt (35). Regular yogurt is made from full-fat milk containing approximately 3.3% of milk fat (35). However, non-fat and low-fat yogurt is produced from partially skimmed milk, skim milk, and low-fat milk (36). According to the Codex Alimentarius Commission (37), the minimum level of milk protein, milk fat, sum of microorganisms constituting the starter culture, and labeled microorganisms allowable in yogurt is 2.7, 15%, 10^7^, and 10^6^, respectively.

#### 1.1.3. Classification based on the flavor

Adding different flavors to yogurt can enhance the sensory property and improve consumer appeal (38). Under this category, yogurt can be categorized into flavored yogurt and plain yogurt. The flavor can be added before homogenization or immediately after the homogenization process.

##### 1.1.3.1. Flavored yogurt

Adding flavors to yogurt can enhance its sensory properties and improve consumer appeal (39). Flavored yogurt can contain flavor enhancers, as well as other additives, such as acidity regulators, color, emulsifiers, packaging gases, stabilizers, and sweeteners (40). Few examples of flavor enhancers allowed in flavored yogurts include magnesium gluconate, glutamic acid, (L+)-, monosodium L-glutamate, monopotassium L-glutamate, calcium di-L-glutamate, monoammonium L-glutamate, magnesium di-L-glutamate, guanylic acid, 5′-, disodium 5′-guanylate-, dipotassium 5′-guanylate-, calcium 5′-guanylate, and inosinic acid, 5′-, at a maximum level of GMP (37).

##### 1.1.3.2. Plain yogurt

Plain yogurt is the least adulterated and simplest form of yogurt, with a pure taste. It is made by bacterial fermentation of pasteurized milk to produce its characteristic flavor and texture (41). In its pure form, it provides the richest concentration of calcium, among other yogurt products. The nutritional composition of plain yogurt is close to that of milk from which it is made, providing all the benefits that are associated with fermentation and, at the same time, supplying few calories (42). It is basically unsweetened, containing little to no additives (36). According to Alimentarius (43), additives in plain yogurt are quite limited to stabilizers and thickeners. The inclusion of other additives, such as color, acidity regulators, flavor enhancers, preservatives, sweeteners, packaging gases, and emulsifiers, is not technologically justified in plain yogurt.

#### 1.1.4. Classification based on making style

Yogurts can be classified widely based on their production style. Some of the most important yogurt styles available in the market are briefly described in this section.

##### 1.1.4.1. Greek-style yogurt

Greek yogurt, also known as strained yogurt, is a common type of yogurt that has been strained or drained off its whey. The result is a thicker and creamier product (44). Nelios et al. (45) reported that the removal of yogurt whey has made Greek yogurt a popular type of yogurt that offers numerous essential health benefits. Notably, it contains twice the protein contained in regular yogurt, making it an important protein source for vegans and vegetarians. Furthermore, it can be taken as a snack, as its protein content makes it more satisfying and filling (46). Furthermore, the probiotics present in Greek yogurt help to maintain a good level of beneficial bacteria to keep a healthy gut. Active probiotics can also boost the immune system and prevent the onset of certain diseases (47). The calcium content of Greek yogurt is vital in building healthy bones and teeth and in preventing osteoporosis (12).

##### 1.1.4.2. Balkan-style yogurt

Balkan-style yogurt, also known as set-style yogurt, has a thick texture and is often made in batches. During the production process, pasteurized milk is added to the cultured bacteria, followed by incubation (36). This yogurt style does not permit any stirring for approximately 12 h or more until the desired creaminess and thickness are attained (36). The Balkan-style yogurt can serve many purposes. It is often used as a substitute for sour cream in making Balkan meat-based recipes. In addition, it is used in Mediterranean dishes (such as spanakopita, moussaka, and pita sandwiches) as a salad topping or dressing. Similarly, it can be consumed as yogurt, sweetened with honey, sugar, or chopped fruit (36).

##### 1.1.4.3. French-style yogurt

This yogurt style is made according to the French process and culture. It is also referred to as custard-style yogurt, made by culturing milk directly in the pot. The product has a pudding-like texture (48). It could be flavored with fruit pieces, most commonly blueberries and strawberries, or a mixture of both, which are stirred directly into the mixture. Diverse studies have found it to be an ideal source of protein, iron, and vitamin A (36).

## 2. Yogurt production

The production of yogurt is by the fermentation of milk with bacteria, which forms lactic acid that causes the milk to thicken and sour (49). The production of yogurt is a multi-step process that involves heating milk, adding a starter culture, and permitting the mixture to ferment. The final product is a thick and creamy food that is rich in probiotics, protein, and calcium (50). Cow’s milk is mainly used to produce yogurt. However, Tamime and Robinson (51) reported that goat, sheep, and buffalo milk can also be a suitable replacement for cow’s milk, and in some cases, powdered milk can be a viable alternative. The milk is usually homogenized to evenly distribute the fat content and pasteurized afterward to kill any harmful bacteria that might be present. In addition to milk, yogurt often contains added sweeteners, such as sugar, corn syrup, or fruit concentrate. These are used to balance the sourness of the yogurt and improve its flavor. Other ingredients that may be added include stabilizers, such as gelatin or pectin, to enhance the mouth feel of the yogurt, and flavorings, such as fruit, spices, and vanilla (52).

### 2.1. Yogurt-making process

**Figure 2:**
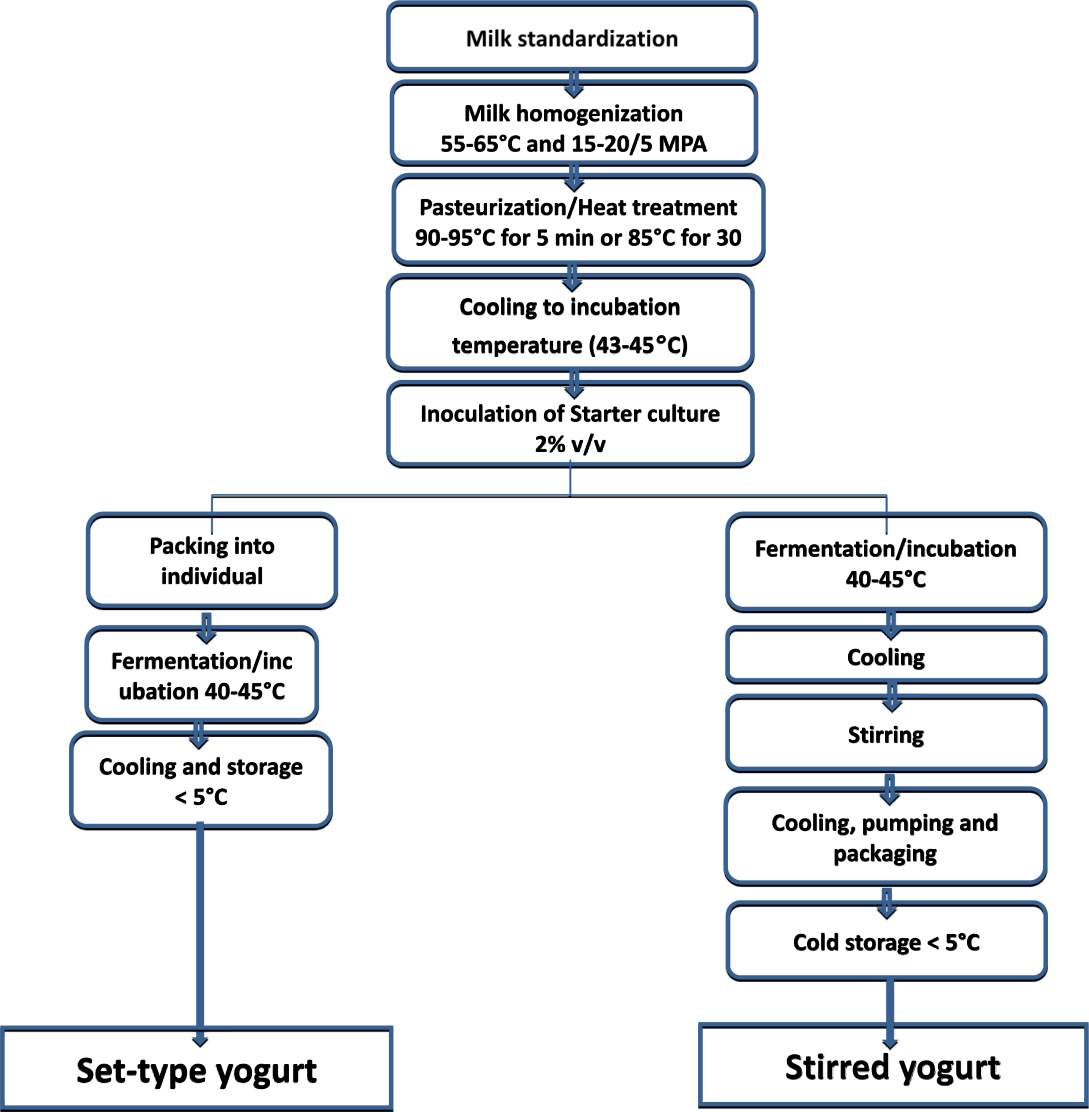
A flowchart of yogurt-making process.

According to Lee and Lucey (53), the main processes involved in yogurt production are the standardization of the fat and protein contents of milk, milk homogenization, heat treatment, incubation and fermentation of milk, cooling, and storage. A starter culture is often added to the milk, and in some cases, diverse additives are added to enhance its nutritional benefits (54). A flow chart of yogurt-making process is shown in Figure 2.

#### 2.1.1. Milk standardization

Based on the desired level of fat content, skim milk and cream are often mixed with milk. The regulations stated by Codex specify that the minimum amount of milk protein allowed in yogurt production is 2.7%, except for concentrated yogurt, where the minimum protein content can be up to 5.6% upon concentration. In addition, the maximum allowable fat content in yogurt is 15% (55). Membrane processing and evaporation under a vacuum are the concentration processes that can increase the total solid content of milk. A study conducted by Iftikhar et al. (56) reported a unique production of set-type yogurt by standardizing milk with 15% total solid and 3.5% fat. The milk powders, whey protein concentrate, non-fat dry milk, or milk protein concentrate can be mixed using a powder dispersion unit (53). Furthermore, in a recent study, Garczewska-Murzyn et al. (57) investigated the impact of buttermilk and skimmed milk powder on the properties of low-fat yogurt. They concluded that there was no difference between the fatty acid profile of yogurt enriched with buttermilk powder and skimmed milk powder.

The texture, appearance, mouth feel, consistency, and viscosity of yogurt can be improved by adding stabilizers (such as gelatin and pectin) to the milk base. Stabilizers can also prevent the whey off of yogurt and improve its consistency (51). In a study conducted to investigate various parameters, such as the water holding capacity, physicochemical properties, low-field nuclear magnetic resonance, texture, and microstructure of set-type yogurt added with commercial stabilizers (gellan gum, sodium alginate, and konjac gum), the results obtained indicated that yogurt with an ionic polysaccharide of gellan gum had improved desirability (58).

#### 2.1.2. Milk homogenization

Homogenization is highly important, especially for yogurt with a high-fat content. Milk is often homogenized at a regulated pressure of 10–20 MPa (first-stage pressure), 5 MPa (second-stage pressure), and a temperature between 55 and 65°C (53). When milk is homogenized at such pressure and temperature, the milk fat globules will disorient into smaller fragments and the surface area will increase. Yogurt homogenization prevents creaming or fat separation, during the process of fermentation or storage. Moreover, it increases whiteness, reduces whey separation, and improves yogurt consistency (53). Upon homogenization, whey proteins and caseins form a layer of fat globules that increases the number of structure-building components in yogurt from homogenized milk (53). Recent studies have investigated the use of ultrahigh-pressure homogenization (200 or 300 MPa) for yogurt production. The results indicated a better firmness and water-holding capacity as compared to conventional homogenization (59, 60). Similarly, Ergin and Küçükçetin (61) demonstrated that different homogenization sequences and milk temperature altered the physicochemical properties of stirred yogurt.

#### 2.1.3. Heat treatment

Milk heat treatment is performed with the sole aim of destroying microorganisms that may alter the attributes of yogurt. Heat treatment can remove dissolved oxygen to prevent heat-induced reactions with starter cultures, as yogurt starter cultures are sensitive to dissolved oxygen (53). Applying heat treatment tends to increase the viscosity and texture of milk in preparation for yogurt production. These structural changes are often a result of a denatured whey protein. Similarly, the acid-induced gelation of yogurt depends on the level of the isoelectric pH of the whey protein (62). The combination of temperature and time for heat treatment in the yogurt industry is 90–95°C for 5 min or 85°C for 30 min (53). Heating milk at a very high temperature lasts between 100 and 130°C for 4 to 16 s, while heating it at an ultrahigh temperature of 140°C should last approximately 4 s (63).

#### 2.1.4. Fermentation

The pre-heated milk should be allowed to rest after heat treatment, to reduce its temperature because the temperature of the milk base must be comfortable for the growth of the starter culture. The optimum temperature of thermophilic bacteria is often approximately 40–45°C (53). Milk fermentation by bacteria converts lactose into lactic acid, which, in turn, reduces the pH of the milk. It is of note that the milk pH decreases from 6.7 to 4.6 during milk acidification. Milk gelation can then occur at a pH of 5.8 to 5.44 (for milk treated with high heat) (53).

A study by Bowler et al. (64) reported the use of ultrasonic sensors in combination with machine learning models to monitor the fermentation of milk. The study suggested that a non-transmission-based and convolutional neural network with a short-term memory layer is the most accurate method for the monitoring of yogurt fermentation. Another study, conducted to determine the effect of single and combined bacteria on the quality of yogurt, suggested that binary probiotics might increase the production of pyruvic acid and L-glutamic acid, enhancing the antioxidant capacity and probiotic of yogurt (65).

##### 2.1.4.1. Microorganisms used as starter culture in yogurt production

*Streptococcus thermophilus* and *Lactobacillus delbrueckii* subsp. *bulgaricus* are key yogurt lactic acid-producing bacteria (LAB) that work together in a mutualistic relationship to ferment yogurt (66). These bacteria can grow independently in milk; however, combining them is crucial for improving the textural properties of yogurt by producing exopolysaccharides and providing health benefits to consumers (66, 67). Horiuchi and Sasaki (68) reported that yogurt fermentation with *Lactobacillus delbrueckii* subsp. *bulgaricus* and *S. thermophilus*, which are facultative anaerobes, proceed effectively even when oxygen is present, as these bacteria can thrive under both aerobic and anaerobic conditions. This protocooperation and mutualistic association involve competition for milk nutrients and the exchange of metabolites (67, 69).

###### 2.1.4.1.1. *Lactobacillus delbrueckii* subsp. *bulgaricus*

*Lactobacillus delbrueckii* subsp. *bulgaricus* is a safe probiotic that confers health benefits to human beings (70). It thrives optimally at a temperature range of 43–46°C and can associate with other bacteria, such as *Streptococcus thermophiles*, to ferment milk (71). *Lactobacillus delbrueckii* subsp. *bulgaricus* ferments carbohydrates to produce lactic acid as its primary by-product (72). The lactic acid produced consists of 95% D-lactic acid and 0.5% L-lactic acid (66). A previous study to examine the genomic nucleotide composition of *Lactobacillus delbrueckii* subsp. *bulgaricus* revealed that the guanine and cytosine contents of the bacteria are in the proportion of 49–51% (73). Another study comparing the genomic composition of *Lactobacillus delbrueckii* subsp. *bulgaricus* with other *Lactobacilli* species found that *Lactobacillus delbrueckii* subsp. *bulgaricus* had a significantly higher G + C content than others in its genus (74). These high guanine and cytosine contents in the third codon position contribute to its elevated genomic structure (74).

In addition, *Lactobacillus delbrueckii* subsp. *bulgaricus* possesses a broad amino acid and proteolytic transport system that enhances its metabolic efficiency in protein-rich environments (75, 76). It also exhibits preferential growth in media rich in lactose and encodes various metabolic pathways (partial pathways) to support its metabolic efficacy. Several studies on the growth conditions of *Lactobacillus delbrueckii* subsp. *bulgaricus* have reported that it can be selectively isolated and identified from the environment or food products using well-defined growth media, such as agar (with an optimum pH of 4.6), a temperature of 43°C, pH-modified MRS (deMan, Rogosa, and Sharpe), and an anaerobic environment (77–79).

###### 2.1.4.1.2. Streptococcus thermophilus

*Streptococcus thermophilus* is a gram-positive bacterium known for its homofermentative metabolism, where it utilizes the Embden–Meyerhof pathway to ferment lactose, ultimately yielding lactate as the end-product (80). It can thrive at an optimal growth temperature of 42°C, and is classified as an aerotolerant anaerobe, belonging to the salivarius group, which includes *S. salivarius* and *S. vestibularis* (81). *S. thermophilus* is uniquely positioned in the food industry as the only *Streptococcus* species used extensively. It has been consumed by humans for centuries without causing any diseases, earning recognition as a Generally Recognized As Safe (GRAS) bacterium by the Food and Drug Administration (FDA) (82). In yogurt production, it is a fundamental starter bacterium and ranks as the second most important species among industrial LAB. It plays a central role in rapid acidification through lactic acid production. Additionally, *S. thermophilus* contributes to developing secondary fermentation products, such as formate, acetaldehyde, and diacetyl, enhancing the aromatic and textural qualities of fermented products (83). Beyond its traditional role in yogurt preparation, *S. thermophilus* contributes to the production of various cheese varieties, including Emmental, Camembert, Brie, Mozzarella, and Parmesan (84, 85).

##### 2.1.4.2. Role of fermentation in generating bioactive peptides

Fermentation is a biotechnological and viable technique for developing new products with improved physicochemical, nutritional, and sensory qualities (86). The significance of fermentation has expanded beyond food preservation, as it plays an important role in the generation of bioactive peptides. Bioactive peptides are essential health-promoting food ingredients with the potential to enhance human wellbeing and mitigate the risk of diseases (87). Manzoor et al. (88) reported that bioactive peptides have multifaceted biological activities, including antimicrobial, antioxidant, immunomodulatory, anticancer, antihypertensive, and antidiabetic effects. The action of fermentation on the hydrolysis of protein to liberate bioactive peptides from their original protein structure has been widely reported (89, 90). Different fermenting microorganisms can produce distinct bioactive peptides due to variations in their enzyme profiles and metabolic activities. Therefore, the choice of microorganisms in the fermentation process can influence the type and quantity of bioactive peptides generated. For instance, lactic acid bacteria are commonly used in dairy fermentation and can produce bioactive peptides with potential immunomodulatory properties (91). Taniguchi et al. (92) published a study on the properties of positively charged peptides derived from fermented soybean Natto. They concluded that the isolated peptides possessed angiotensin I-converting enzyme (ACE-I), antimicrobial, anticancer, antihypercholesterolemic, lipopolysaccharide (LPS)-neutralizing, and angiogenic activities. Chen et al. (89) reported that *Lb. helveticus* liberated the following peptides (VPP, IPP, LKP, ALPM, PGPIHD, VAGTWY) to inhibit the action of ACE-1. Solieri et al. (93) also disclosed the role of fermentation with *Lb. casei* PRA205 or *Lb. rhamnosus* PRA331 in the liberation of VPP and IPP having ACE-1 activity. Similarly, Rutella et al. (94) reported the biological activity of *Lb. casei* PRA205 or *Lb. rhamnosus* PRA331 in liberating peptides with antihypertensive and antioxidative activities.

#### 2.1.5. Cooling

At the desired pH of approximately 4.6, yogurt is to be partially cooled at a temperature of approximately 20°C. Cooling must occur before the addition of flavoring and fruit ingredients. Oftentimes, the yogurt is chilled to above 5°C to reduce any further acid development. In the case of set-type yogurt, it is transferred directly to cooling tunnels. However, stirred-type yogurt is first agitated in the jacketed fermentation vat, before shearing and smoothening by sieves or a high-shear device (53).

## 3. Additives in yogurt and their functions

The Codex Alimentarius Commission defined food additives as any substance that is neither food nor food ingredients that is added to food to serve technological functions in its processing, manufacturing, and preparation (95). In general, yogurt additives are a subset of edible raw materials for yogurt processing, manufacturing, treatment, and preparation (96). The inclusion of additives in yogurt formulation is intended to increase its sensory and technological properties. Thus, the functional properties the additives confer on the yogurt may not be possible in their absence (96).

Additives can be added to yogurt either as fresh materials, such as vegetables and fruits, or dried and powdered extracts (97). Similarly, they can be added during or after pasteurization and fermentation. It is of note that the additive does not only improve the health benefits and chemical composition of yogurt, but it also improves its technological and organoleptic properties, such as color, microstructure, texture, and other sensory properties of the yogurt (19, 98–100). For instance, some natural additives and materials, such as vanilla and strawberry, can enhance the flavor and health-promoting properties of the yogurt, such as antidiabetic, antioxidant, antimicrobial, anti-obesity, and anticancer activities (31, 98, 101–105).

The Joint FAO/WHO Expert Committee on Food Additives (JECFA) has the task of evaluating the potential health and safety risks posed by food additives by determining the acceptable daily intake (ADI) for these additives. However, the choice of whether to consume food containing a specific additive is left to the consumers (106). The list of some of the additives classes approved by the Codex Alimentarius Commission is presented in Table 1.

**Table 1:** List of Codex-approved yogurt additives, including their functions and maximum allowable level.

Additive class	Functions	Codex-approved additive in yogurt	Maximum level
Acidity regulators	Controls and adjusts the pH level of yogurt to maintain the desired level of tartness, texture, and flavor while ensuring the stability and safety of the product during production and storage.	Tartaric acid L(+)- Monosodium tartrate Sodium L(+)-tartrate Monopotassium tartrate Dipotassium tartrate Potassium sodium L(+)-tartrate	2000 mg/kg
Color	Enhance the visual appeal of yogurt by adding or intensifying its color.	Curcumin Riboflavin, synthetic Riboflavin 5′-phosphate, sodium Tartrazine Quinoline yellow Sunset yellow FCF Carmines	100–300 mg/kg
Emulsifiers	Evenly disperse fat throughout the yogurt to improve its texture and creaminess while preventing fat separation.	Polyoxyethylene (20) sorbitan monolaurate Polyoxyethylene (20) sorbitan monooleate Polyoxyethylene (20) sorbitan monopalmitate Polyoxyethylene (20) sorbitan monostearate Polyoxyethylene (20) sorbitan tristearate	3,000 mg/kg
Flavor enhancers	Improve the taste of yogurt by intensifying natural flavors, masking undesirable off-notes, or creating unique and appealing flavor profiles.	Magnesium gluconate Glutamic acid Monosodium L-glutamate Monopotassium L-glutamate Calcium di-L-glutamate Monoammonium L-glutamate Magnesium di-L-glutamate Guanylic acid, 5’	-
Stabilizers and thickeners	Maintain a consistent yogurt texture by preventing the separation of whey and fat while giving yogurt its desired thickness.	Calcium carbonate Trisodium citrate Gellan gum Konjac flour Pectins	-
Sweeteners	Increase the taste and sensory qualities of yogurt, making it more palatable and appealing to consumers	Sorbitol Mannitol Acesulfame potassium Aspartame Cyclamates Isomalt (Hydrogenated isomaltulose) Saccharin	GMP; 100–1,000 mg/kg

## 4. The influence of food additives on the design of yogurt

Food additives play an important function in the design of yogurt and its sensory properties. The lactic acid produced during milk fermentation contributes to the sour taste, texture, and flavor of yogurt (107). However, to satisfy the consumer’s need for specific sensory and functional properties, food additives are added. The addition of these additives can either negatively or positively impact the original nature of the yogurt (108). The impacts of some specific additives on yogurt’s design are discussed in the following sections.

### 4.1. The impact of stabilizers on yogurt design

Fermentation of milk by bacteria culture causes the milk protein to coagulate, thereby forming gel-like substances. However, stabilizers are used to make the yogurt more stable by preventing the separation of milk solids and liquid whey (109). Saadi et al. (110) reported that adding stabilizers to yogurt increases its texture, appearance, and overall sensory qualities. These stabilizers can also improve consumers’ acceptability and positively impact the durability of yogurt (111). In another study by Eze et al. (112), it was revealed that increasing the concentration of stabilizers resulted in an increase in the protein, total solid, sugar, total titratable acid, ash, and fat content of the yogurt while decreasing its moisture content.

Despite the benefits that stabilizers offer to yogurt’s shelf life and durability, its synthetic form has become a subject of controversy. Synthetic stabilizers, such as polyethylene oxide polymers, acrylate copolymers, and polyethylene oxide polymers (Polyox) derived from chemical combinations, are considered the strongest stabilizers. However, their addition to foods is undermined by certain drawbacks associated with them, such as increased environmental pollution during synthesis, potential harm to consumers due to toxicity, and inferior biocompatibility in contrast to naturally sourced alternatives. Some studies have also identified that some modified stabilizers, such as carrageenan, may be linked to inflammation, digestive issues, and other health problems (113). In contrast to synthetic stabilizers, natural food thickeners, such as starches and natural gums, are hydrocolloid polysaccharide polymers with the capability to stabilize the emulsion and form a gel. Natural gum has advantages over synthetic stabilizers as they are less expensive, chemically inert, biodegradable, and non-toxic. Natural gum and starches have also been reported to be suitable prebiotic substrates for probiotic genera, such as the genus Bifidobacterium (114). Hussein et al. (115) also reported the use of chickpeas as a stabilizer, antioxidant, prebiotic, and thickening agent in yogurt. Their findings indicated that the chickpea stabilized yogurt and enhanced the population of probiotic bacteria in bio-yogurt during storage.

It is important to note that using too little stabilizer may not meet functional requirements, while excessive amounts can lead to undesirable appearance and texture, such as an unappealing mouthfeel, surface sheen, and rubbery texture. Therefore, precise control over stabilizer concentration is vital for producing a high-quality yogurt with high sensory scores for viscosity, creaminess, mouthfeel, and consumers’ acceptability (116).

### 4.2. The impact of sweeteners on yogurt design

Although yogurt has a natural sour and tangy taste, which is contributed by the bacteria fermentation of milk, sweeteners are employed by manufacturers to further improve its taste (117). The sweetener can be synthetic, natural, or sugar substitutes (118). These sweeteners, regardless of their origin, have their impacts on yogurt designs. Some examples of synthetic sweeteners are aspartame, saccharin, and sucralose (119). They are often present in high concentrations and are aimed at adding a sweet taste to yogurt. However, on the downside, they can affect the texture and flavor of the yogurt, making it less appealing to some consumers. For instance, aspartame can leave a bitter aftertaste to yogurt, while saccharin can affect the flavor of the yogurt (120).

Natural sweeteners, such as stevia and monk fruit extract, have increased in popularity over the years due to their health benefits and natural origin. A study carried out by Chadha et al. (121) revealed that yogurt sweetened with monk fruit decreased hunger levels as compared to yogurt sweetened with sucrose. The taste and sweetness of these sweeteners differ as a result of their significant mixture and processing method, which may cause difficulties in achieving consistency in yogurt design (122). However, in terms of consumers’ acceptability, Mousa et al. (123) reported that yogurt sweetened with natural additives (stevia) had low acceptability compared with sucrose-sweetened yogurt. In another study that assessed the sensory acceptability of low-fat strawberry yogurt added with different sweeteners, it was found that consumers preferred the yogurt sweetened with sucrose over that sweetened with high-fructose corn syrup or honey (124).

### 4.3. The impact of colorants on yogurt design

The use of colorants in yogurt is regulated by diverse international food safety agencies (125). These regulatory bodies set guidelines for the acceptable levels of colorants in food. The color in yogurt can affect consumer’s perception in several ways, as consumers associate specific colors with certain flavors, such as yellow with lemon and red with strawberry (126). This color and flavor association has greatly impacted consumer preference as the perceived flavor of yogurt may be altered by changes in its color (127). Modified colorants are less favored due to their adverse effects on human health. However, some of the artificial colors, such as blue #1, red #40, and yellow #6 and #5, are still very much in use today due to their affordability and color consistency (128).

Contrary to modified colorants, natural colorants, such as fruit purees and vegetable juices, are often favored due to their perceived health benefits (129). In this context, a study by Patiwit and Anuchita (130) revealed that yogurt with a natural colorant obtained from black rice bran could potentially improve the functional properties and overall qualities of yogurt. Hence, these additives can also serve as antioxidants and prebiotic sources. Furthermore, Vidana et al. (131) supplemented yogurt with blue pea flowers. The result was an intense blue yogurt in the acidic pH range of 3.2–5.2, making it suitable as a natural blue food colorant. Additionally, blue pea flower anthocyanin extract has significant *in vitro* and cellular antioxidant activity, contributing to their potential health benefits.

Consumers’ acceptability is an important aspect to consider when incorporating colorants into yogurt, as it directly influences the overall appeal and market success of the product. In this regard, Murley and Chambers (132) investigated consumer perceptions of the sources of food colorants in different food products, including yogurt, across different nations. Their findings (Table 2) revealed that participants from the United States, the United Kingdom, and Australia shared consistent attitudes toward suitable sources for natural colors. Notably, Australian respondents showed a preference for a wider range of color sources, while American participants were more selective. Additionally, the study found a strong preference for plant-derived additives over those from animals, insects, microbes, or minerals, with fruits, fruit juices, vegetables, and flowers being the most frequently chosen sources for color. These findings align closely with the FDA’s position on natural color additives.

**Table 2:** Acceptability ratings for color sources in natural foods and beverages (including yogurt) among respondents from the US, the UK, and Australia.

Color source	US	UK	Australia
Fruit juice	64%	63%	66%
Juice	76%	76%	80%
Insects	17%	35%	26%
Chemicals	20%	22%	23%
Algae	33%	34%	35%
Vegetables	70%	74%	79%
Meat	23%	22%	25%
Flowers	52%	60%	64%
Beans	38%	35%	38%
Extracts	52%	49%	50%
Vitamins	23%	17%	19%
Minerals	30%	29%	32%
Animal skins or bones	12%	13%	15%
Roots	44%	46%	49%
Food dyes	36%	42%	45%
Grains	26%	21%	27%
Clay	14%	16%	18%
Seaweed	41%	52%	56%
Beneficial microorganisms	10%	11%	11%
Leaves	42%	47%	55%
Bark	26%	26%	28%

Adapted from Murley and Chambers (132).

### 4.4. The impact of flavorants on yogurt design

Flavorants are added to yogurt to enhance its taste and aroma. They affect the final taste and appearance of the yogurt (133, 134). The majority of synthetic flavoring agents are derived from petroleum, consisting of numerous volatile chemicals (135). Some synthetic flavoring agents are considered unsafe. For instance, sodium nitrate is a carcinogenic compound, while artificial vanilla flavor may induce allergic reactions and reduce the activity of the liver enzyme dopamine sulfotransferase by 50% (136). In contrast, natural flavors can be derived from natural sources, such as herbs, fruits, and spices (137). Some of the natural flavoring agents, such as chlorogenic acid (from green bean coffee), paprika, vanilla extract, and butter flavor have been reported to possess antidiabetic, anti-aging, anti-obesity, anti-inflammatory, and anticancer activities (138–140).

The addition of flavorants in yogurt impacts the sensory properties (such as aroma, taste, and appearance) and overall consumers’ acceptability (108). In Table 3, similar to yogurt colorants, Murley and Chambers (132) explored consumer perceptions of yogurt flavor sources across various countries. The study concluded that Australians tended to choose more options for flavor sources, while Americans were the least likely to do so. This might suggest that Americans are more specific in their preferences for yogurt flavor additives, or that Australians are more receptive to a variety of natural additive sources. The most commonly selected sources for flavor include fruits, fruit juices, vegetables, and flowers, aligning with the FDA’s definition of natural flavor.

**Table 3:** Acceptability ratings for flavor sources in natural foods and beverages among respondents from the US, the UK, and Australia.

Flavor source	US	UK	Australia
Fruit juice	68%	65%	68%
Juice	80%	79%	81%
Insects	17%	24%	23%
Chemicals	9%	9%	11%
Algae	29%	32%	33%
Vegetables	72%	76%	81%
Meat	42%	42%	49%
Flowers	45%	50%	57%
Beans	49%	49%	54%
Extracts	46%	42%	43%
Vitamins	26%	21%	20%
Minerals	31%	32%	33%
Animal skins or bones	21%	25%	27%
Roots	49%	48%	56%
Food dyes	11%	13%	14%
Grains	46%	39%	46%
Clay	9%	11%	12%
Seaweed	42%	55%	59%
Beneficial microorganisms	10%	14%	15%
Leaves	41%	45%	54%
Bark	26%	25%	28%

Murley and Chambers (132).

## 5. Effect of additives on the rheological properties of yogurt

An important factor in determining yogurt consistency, texture, and overall quality is its rheological properties (141). Certain factors, such as milk type, starter culture, the concentration of stabilizers or thickeners added, production process, packaging, and storage, significantly affect yogurt’s rheological properties (97). Thickeners, such as gum arabic, pectin, and carrageenan, are used to improve the mouthful, viscosity, and stability of the yogurt. Pectin, as a gelling agent, can be obtained from apple pomace or citrus peels using a hot diluted mineral acid at a low pH. The extract can then be filtered to obtain the precipitated pectin using isopropanol or solvent ethanol (142, 143). Gums are stabilizers of suspension and emulsions. Carrageenan, which is a common hydrocolloid, is obtained from red seaweed, particularly from Irish moss, and is used to improve the stability and texture of yogurt (128). All these aforementioned thickeners can play a vital role in impacting the rheological properties of yogurt, including its yield stress, flow behavior index, and consistency coefficient (144, 145). Yield stress is the minimum stress needed to initiate the flow in a material. Adding thickeners to yogurt can increase its yield stress, thereby making it more stable (146). The flow behavior index characterizes and defines the rheological nature of the yogurt, while the consistency coefficient measures yogurt’s flow resistance (144, 145). The flow index and consistency coefficient can be defined following the Freundlich model, from the association between the shear strain rate and the shear stress (146).

Himashree et al. (147) formulated yogurt supplemented with modified starches and seaweed extracts. Their results showed that adding thickeners improved the handling properties, mouthfeel, and stability characteristics of the yogurt. However, factors such as temperature, pH, shear, and ionic strength affect the functionality of the thickeners and, therefore, must be optimized during formulation by food processors. Similarly, Szołtysik et al. (25) conducted a study to monitor the rheological and physicochemical properties of yogurt enriched with honeysuckle berries and resistant starch (both combined and individually). They reported that the additives did not pose radical changes in the yogurt’s rheological parameters, physicochemical properties, and acidity. In addition to adding thickeners to yogurt, stabilizers, such as gelatin and sodium alginate, are added to yogurt to improve its texture and stability (148). Gelatin, a protein that is extracted from animal collagen, is known for its gelling properties (149). On the other hand, sodium alginate, a polysaccharide obtained from brown seaweed, was used as a stabilizer in the commercial production of yogurt (150). Furthermore, Khubber et al. (27) documented that adding low-methoxyl pectin improved the rheology of low-fat set yogurt. Pertaining to a possible mechanism for the improved rheology, the authors suggested that casein interactions with anionic low-methoxyl pectin, due to electrostatic bond formation at a reduced pH (4–5), might have resulted in a compact protein gel structure-packing. According to the authors, this, along with the hydrated hydrocolloid’s filling action, could have led to yogurt’s solid gel-like property with more elasticity, instead of a viscous flow.

## 6. Nutraceutical benefits of adding natural and modified additives in yogurts

Yogurts formulated with natural and modified additives have several nutraceutical benefits, including enhancement of bioactive compounds, antioxidant, antihyperglycemic, and antihypertensive effects, as reported in the literature. de Carvalho et al. (151) reported that adding freeze-dried stevia extract improved the antioxidant capacity of yogurt. In another study, Wang et al. (152) investigated the effects of adding thermally processed mulberry leaf extract on the bioactive constituents, antioxidant, and α-glucosidase inhibitory activities of yogurt. Their findings revealed that mulberry leaf extract addition resulted in a higher total anthocyanin, flavonoid, and phenolic contents and improved the DPPH^*^ scavenging activity of the yogurt. Their study further indicated that yogurt fermented with 15% roasted mulberry leaf extract displayed the strongest α-glucosidase inhibitory effect when compared with yogurt fermented with 15% native (untreated) and steamed mulberry leaf and extracts. An increase in the antioxidant activity of roasted foods could be attributed to the Maillard reaction products formed during roasting, as the Maillard reaction products exhibit antioxidant activity (153, 154).

Furthermore, Wajs et al. (155) investigated the effects of plant-based additives on the functional, physicochemical, sensory properties, and microbiological attributes of yogurt. Their findings revealed that yogurt supplemented with plant additives was rich in health-promoting compounds, such as phenolic compounds, minerals, fatty acids, vitamins, and fibers. The phenolic compounds found in the additives can protect the body from some diseases, promote cardiovascular health, protect the brain cells, and support digestive health. Adding ginger extract to yogurt has also proven to improve its functional properties. Rifky et al. (156) reported that ginger contains phytochemicals and polyphenolic compounds that express high antioxidant activity as well as therapeutic and preventive properties. Still on antioxidant activity, fruit powder of sun-dried white mulberry, added at 2, 4, and 6% levels, improved the antioxidant potential of buffalo milk-based yogurt (21). The increase in antioxidant potential following sun-dried white mulberry fruit powder addition was attributed to the abundant phytochemicals present in white mulberry. Earlier, Devi et al. (157) reported some antioxidant phytochemicals in white mulberry, including ascorbic acid, vitamin B1, β-carotene, folic acid, folinic acid, quercetin, isoquercetin, tannins, saponins, and flavonoids. Aside from the antioxidant effect of these phytochemicals in mulberry, an elevated antioxidant capacity typical of fermented milk products is ascribed to lactic acid bacteria’s proteolytic action and the concomitant release of bioactive peptides (158).

Honey is a well-pronounced functional additive in the production of healthy yogurt. Sarkar and Chandra (159) reported that the medicinal and nutritional value of honey, in conjunction with the presence of oligosaccharides, has made it a viable, functional additive in yogurt production. Another study by Ahmed et al. (97) illustrated that yogurt made from plant-based milk, such as soybean milk, added with functional ingredients enhanced the biological activities of yogurt. However, this improvement can be widely affected by the milk source, ingredient type, and duration of storage. In another study, the addition of grape pomace powder and peel powder of wild pomegranate increased the phenolic and flavonoid constituents of the yogurt (160). Szołtysik et al. (161), in a study to evaluate the effect of *Rosa spinosissima* fruit extract on lactic acid bacteria growth and other yogurt parameters, revealed that the addition of the fruit extract improved the microflora counts and antioxidant properties of the yogurt.

In another study, Dabour et al. (162) formulated a yogurt supplemented with brans or dietary fibers extracted from either rice or wheat. They evaluated its effect on the serum lipids and hepatic function in hypercholesterolemic male rats. Their findings revealed that dietary fibers or brans reduced serum glucose and improved the lipid profile and hepatic antioxidant status of the rats. Furthermore, Phoem et al. (163) highlighted that utilizing *Lactobacillus plantarum* combined with *Eleutherine americana* oligosaccharide extract as an additive in yogurt increased antibacterial effectiveness against enteropathogenic bacteria. Furthermore, Shori (164) evaluated the proteolytic, antioxidant, and α-amylase inhibitory activities of yogurt added with coriander leaves and cumin seeds aqueous extracts at varying levels (5, 10, 15, and 20 g/100 ml). The study concluded that at all concentrations, the addition of coriander leaves and cumin seeds extracts enhanced the yogurt’s antioxidant and α-amylase inhibitory activities, suggesting an antihyperglycemic effect.

Several other formulations have been reported to improve the health-promoting properties of yogurt, so as to meet the growing demand for healthy yogurt (31, 165–167). A list of some other natural additives recorded to confer nutraceutical benefits on yogurt and improve its overall quality is presented in Table 4.

**Table 4:** List of natural additives, their evaluated parameters, and outcome.

Additives used	Evaluated parameters	Outcome	Reference
Pumpkin (control), carrots, green peas, andzucchini—puree	Physicochemical aspects (pH level, acidity, and lactic acid content) Syneresis rate Color analysis using instrumentation Quantification of total solids, ash, and dietary fibers Measurement of total antioxidant capacity, ascorbic acid, total phenolic content, and total carotenoids Evaluation of texture parameters (firmness, cohesiveness, consistency, viscosity index) Sensory evaluation covering appearance, texture, aroma, taste, color, flavor, sensory acidity, and overall impression	Higher counts of beneficial bacteria (*Streptococcus thermophiles* and *Lactobacillus delbrueckii* subsp. *bulgaricus*) from the vegetable-fiber enriched yogurt. Presence of fibers, phenolic compounds, and organic acids in the vegetables enhanced the growth of lactic acid bacteria. Increased antioxidant capacity in pumpkin yogurt due to higher total phenolic, ascorbic acid, and total carotenoid contents. Potential increase in essential nutrients, such as vitamins and minerals from vegetable supplementation	(168)
Pineapple—peel powder	Physicochemical attributes (including pH value, water holding capacity) Texture-related aspects Rheological characteristics (such as texture and hardness) Sensory properties (specifically color) Microstructural traits	Improved fiber content Increase in syneresis, pH value, firmness viscosity, and color intensity	(169)
Basil gum from basil seeds	Physicochemical parameters (pH value, total titration acidity) Viscosity measurement Color analysis using instrumental methods Syneresis evaluation Quantification of fat and moisture content Measurement of total phenol, flavonoid, and antioxidant activity Sensory assessment (covering appearance, color, flavor, texture, sourness, and overall acceptability)	Elevated levels of total content of phenolics and flavonoids Enhanced physicochemical characteristics Improved antioxidant activity and sensory attributes	(170)
Blue pea flower (*Clitoria ternatea* L.)	Physicochemical properties DPPH antioxidant levels Sensory (color)	Elevated antioxidant potential Amplified color vibrancy	(171)
*Hibiscus sabdariffa* L. flowers	Physicochemical analysis (dry matter, ash, protein, pH, titratable acidity, water-soluble dry matter, water activity, syneresis, antioxidant acidity, and total phenolic content) Texture assessment (viscosity) Sensory evaluation (appearance, texture, aroma, taste, color, and overall acceptability) Microbial examination (*Lactobacillus delbrueckii* subsp. *bulgaricus*, *Streptococcus thermophiles*, *Escherichia coli*, and *Staphylococcus aureus*).	Enhancements observed in: Titratable acidity Total solids Ash content Antioxidant activity	(172)

## 7. Conclusion and future perspective

With more concerns being raised on the use of synthetic additives in yogurt production, studies have illustrated the potential rheological and nutraceutical benefits of natural and modified additives in providing healthy yogurt for consumers. With continuous research and innovation, the use of natural and modified additives in yogurt formulation will enhance its health-promoting properties, while improving the overall quality. Hence, further research, optimizing the proportions of the various natural and modified additives incorporated in yogurt formulation to enhance its overall quality, is suggested. Similarly, investigating advanced delivery systems or encapsulation techniques for natural and modified additives, to improve their stability and bioavailability in yogurt, is imperative.

## Author contributions

AB: Writing – original draft, Writing – review & editing. EI: Conceptualization, Writing – review & editing. WA: Writing – review & editing. EA: Writing – review & editing.
